# Cognitive interventions for adults with chronic kidney disease: protocol for a scoping review

**DOI:** 10.1186/s13643-020-01320-x

**Published:** 2020-03-17

**Authors:** Janine F. Farragher, Katherine E. Stewart, Tyrone G. Harrison, Lisa Engel, Samantha E. Seaton, Brenda R. Hemmelgarn

**Affiliations:** 1 Department of Community Health Sciences, Cumming School of Medicine, University of Calgary, Rm G236, 3330 Hospital Drive NW, Calgary, AB T2N 4Z6 Canada; 2 Rehabilitation Sciences Institute, University of Toronto, Toronto, Canada; 3 Department of Medicine, University of Calgary, Calgary, Canada; 4 Department of Occupational Therapy, University of Manitoba, Winnipeg, Manitoba Canada; 5 O’Brien Institute for Public Health, Cumming School of Medicine, University of Calgary, Calgary, Canada; 6 Libin Cardiovascular Institute of Alberta, Cumming School of Medicine, University of Calgary, Calgary, Canada

**Keywords:** Cognition, Chronic kidney disease, End-stage renal disease, Dialysis, Scoping review

## Abstract

**Background:**

Cognitive impairment is a common and frequently under-recognized complication of chronic kidney disease (CKD). Although there is extensive literature on cognitive interventions that can ameliorate cognitive impairment or associated negative outcomes in the general literature, the breadth and characteristics of cognitive interventions that have been studied in people with CKD are currently unclear. The objective of this scoping review is to identify and describe the literature on cognitive interventions for adults with CKD, including end-stage kidney disease (ESKD).

**Methods:**

A scoping review following Joanna Briggs Institute methodology will be conducted. With assistance from an information specialist, we will search 5 electronic databases (MEDLINE [OVID], EMBASE, PsycINFO, Cochrane Central Register of Controlled Trials, and CINAHL Plus) using search terms that represent the target population (CKD) and concept (cognition), and conduct backward citation searching for additional literature. Eligible sources will be primary research studies (quantitative or qualitative) that investigate any intervention targeting cognition in adults (≥ 18 years) with CKD or ESKD, including those treated with dialysis. We will extract data about characteristics of interventions (e.g., type, underlying theory, design, location, and provider), populations (e.g., stage of CKD, age, sex, and type of cognitive impairment), and studies (e.g., authors, location, design, and reported findings). Article screening and data extraction will be performed by two to three reviewers. Data will be analyzed using descriptive statistics and narrative syntheses to characterize the literature on cognitive interventions for people with CKD.

**Discussion:**

This study will provide a comprehensive overview of the cognitive interventions that have been studied for people with CKD. It will help identify research gaps within this population (e.g., types of interventions that have yet to be investigated; best practices in cognition research that have not been implemented) and inform the direction of future research in this field.

## Background

Cognitive impairment is a highly prevalent and frequently under-recognized complication of chronic kidney disease (CKD) [1, 2]. The concept of cognitive impairment includes lower than average performance or decline in domains of cognition (e.g., attention/orientation, awareness and insight, memory, reaction and/or processing, executive functions, reasoning, judgment/decision-making, and planning). The Chronic Renal Insufficiency Study found that reduced kidney function is independently associated with worse cognition in most cognitive domains, after adjustment for demographic and clinical factors [2]. Multiple domains of cognition have been found to be affected by CKD, including orientation, attention, language, executive functioning, and reasoning [3, 4]. Evidence from prospective cohort studies suggest that cognitive impairment worsens as CKD progresses [3, 5], and becomes particularly prevalent in patients with end-stage kidney disease (ESKD) (i.e., kidney failure where provision of dialysis or transplantation is considered). A study of 676 people treated with chronic hemodialysis found that 71% of participants displayed impaired cognitive function, while 45% were impaired in two or more cognitive domains [6]. Similarly, in a multicenter study of 376 people on hemodialysis aged 55 or older, only 13% were found to have unimpaired cognitive functioning [1] on a comprehensive cognitive testing. Notably, only 4% of those found to have severe cognitive impairment in the study had a documented diagnosis of dementia [1], suggesting that cognitive impairment frequently goes unrecognized in this population.

The mechanism of developing cognitive impairments in CKD is hypothesized to result from a combination of vascular and non-vascular factors [7]. Vascular factors include subclinical cerebrovascular disease in the form of white matter lesions, silent brain infarcts, and microbleeds [7–9] and an increased incidence of stroke [10]. Other vascular risk factors, such as inflammation and oxidative stress, are also hypothesized to contribute to cognitive impairment through mechanisms such as accelerated atherosclerosis and vascular endothelial impairment [7]. Hemodialysis treatment itself has been found to cause an acute reduction in cerebral blood flow during treatment sessions, and may interfere with cognitive processes in people with ESKD undergoing routine hemodialysis sessions [11]. Nonvascular factors including neuronal toxicity from uremia, frequent use of angiotensin-converting enzyme inhibitors, depression, and anemia, have also been identified as possible contributors to cognitive impairment in patients with CKD [7, 12]. Little is known about the impact of cognitive impairment on people living with CKD, although it has been found to be associated with increased need for assistance with peritoneal dialysis [13] and increased mortality [14, 15]. In other chronic disease populations, cognitive impairments have been shown to be associated with poor outcomes including functional decline [16, 17], long-term care admission [18, 19], and mortality [17].

**Fig. 1:**
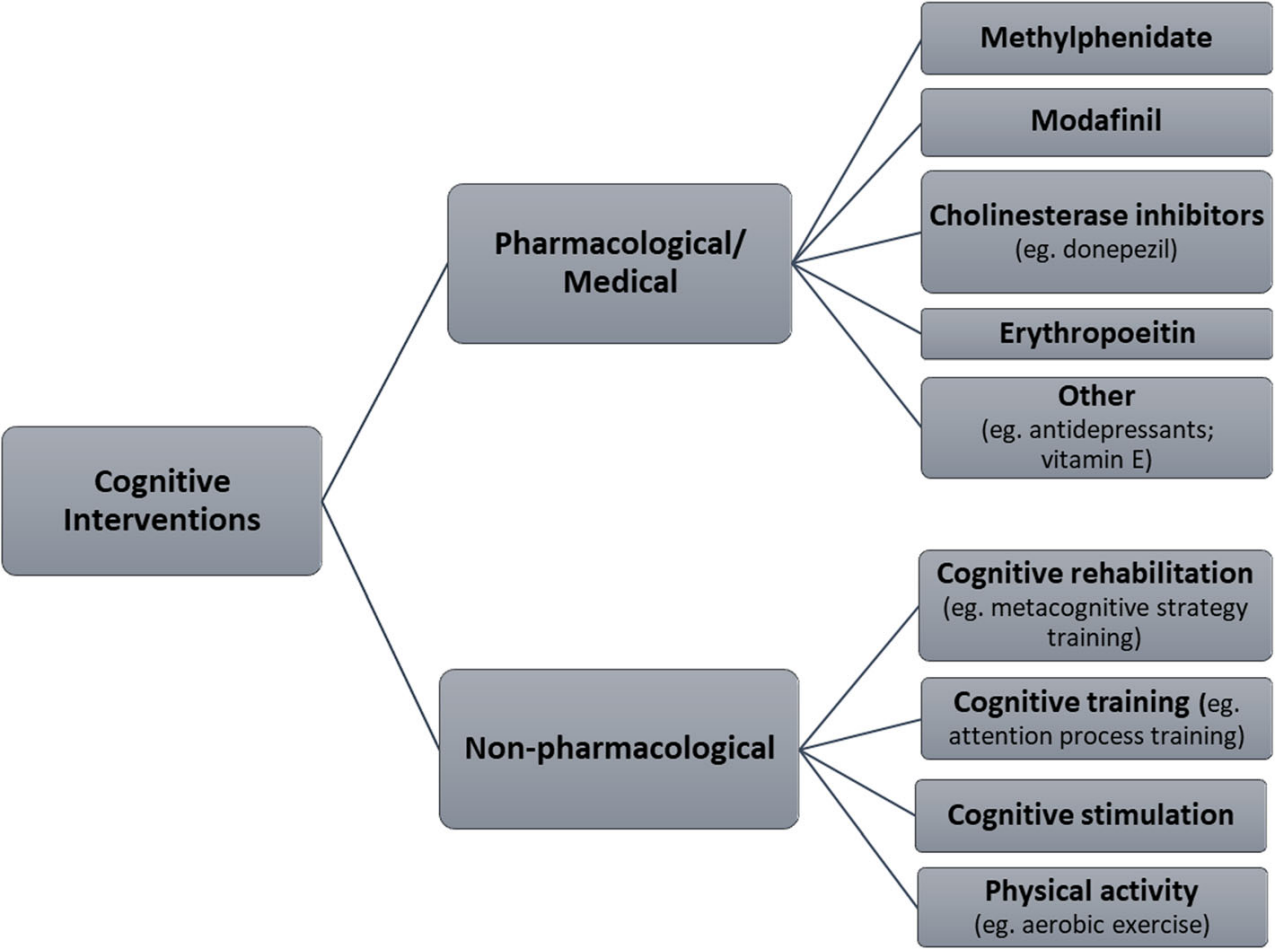
Examples of cognitive interventions used in non-chronic kidney disease populations

There are several pharmacological interventions that have shown potential to ameliorate cognitive impairments or minimize their effects on daily functioning and well-being in other populations (Fig. 1). For example, donepezil is a cholinesterase inhibitor approved for slowing cognitive decline in mild to moderate Alzheimer’s disease [20, 21] that has also been associated with beneficial cognitive effects in people with vascular cognitive impairment [22] and cancer [23]. Modafinil is a central nervous system stimulant that has been associated with improved cognitive performance in healthy subjects [24] and multiple sclerosis [25], although evidence from clinical trials in cancer are inconclusive [23]. Methylphenidate is another central nervous system stimulant that has shown some benefit for cancer-related cognitive impairment in early-phase studies [26]. Erythropoietin is a hormonal treatment for anemia (used commonly in CKD patients) that has been reported to have neuroprotective effects in both animal [27, 28] and human studies [29, 30], while other approaches, such as antidepressant medications for stroke [31] and depression [32], have also been associated with positive cognitive outcomes.

There is also research on non-pharmacological approaches to minimize cognitive impairment or improve daily functioning in chronic disease populations. For example, cognitive rehabilitation has been defined as an “individualized approach to helping people with cognitive impairments (p393)” where the emphasis is on improving functioning in everyday situations, rather than enhancing performance on specific cognitive tasks [33]. Cognitive rehabilitation interventions, which include approaches such as problem solving training and memory notebook training, have shown positive results in diverse clinical populations such as multiple sclerosis [34], Alzheimer’s disease [33], mild cognitive impairment [35], acquired brain injury [36], and other chronic medical conditions [37]. Cognitive training is an alternative approach that instead entails practice on a set of standardized tasks designed to improve specific cognitive functions, such as memory, attention, or language [33]. Attention process training and computerized cognitive training are examples of cognitive training interventions that have demonstrated efficacy at improving performance on neurocognitive tests in some studies [36], although their effects on real-world functioning are less clear. By contrast, cognitive stimulation refers to “engagement in a range of group activities and discussions aimed at general enhancement of cognitive and social functioning (p387)” and reality orientation, and has primarily been used in people with moderate or severe dementia [33]. Exercise-based approaches, such as aerobic exercise training, have also been found to improve cognition and related outcomes in population such as older adults with mild cognitive impairment [38] and stroke [39].

To the best of our knowledge, the literature on cognitive interventions for people with CKD has never been systematically explored and synthesized. It is unclear what pharmacological and non-pharmacological interventions have been studied to address cognitive impairments in CKD, and, to what extent existing research aligns with recommendations from the broader cognitive intervention literature. For example, it has been recommended that pharmacological cognitive interventions should target specific mechanisms of disease, brain injury, accommodation, or recovery using appropriate diagnostic criteria [40], since assumptions about the etiology of cognitive impairments could lead to inappropriate patient selection for studies. Cognitive rehabilitation interventions should be based on a theoretical understanding of the development and organization of human cognitive processes, consider factors such as the presence and severity of impairment and relevant comorbidities, and target specific problem areas that require intervention [37]. The importance of measuring both cognitive process or domain outcomes as well as participation or daily living application outcomes [41] has been noted. Further, it has been argued that studies should use both objective and self-report assessments, as both have benefits and limitations [41], while self-report measures can be prone to bias in inadequately controlled studies, and objective neuropsychiatric measures do not always translate to real-world functioning. Consideration should also be given to stakeholder needs and perspectives in intervention development, as the uptake of cognitive interventions can be affected by factors such as their complexity, adaptability, and the learning needs of both staff and patients.

Scoping reviews are increasingly used to systematically search a body of *literature*, identify knowledge gaps, clarify concepts, and/or investigate research conduct [42]. According to the Joanna Briggs Institute, they can also be conducted as a preliminary exercise prior to a systematic review and are useful “for examining emerging evidence when it is still unclear what other, more specific questions can be posed and valuably addressed” [43]. A scoping review of cognitive interventions for people with CKD will enable characterization of the research activity in this area, discover evidence gaps that warrant further investigation, and identify ways to enhance research in this field moving forward. The objective of this scoping review is to comprehensively identify and describe the literature on cognitive interventions for adults with CKD. Specifically, we aim to answer the following questions:

1. What pharmacological and/or non-pharmacological cognitive interventions have been investigated in people with CKD?
2. What cognitive interventions have been reported to be associated with positive results in CKD, and for which specific population groups and clinical contexts?

## Methods

We will use scoping review methodology consistent with the Joanna Briggs Institute guidance [44] to conduct this review. Additionally, our review will follow the scoping review reporting guidelines of the PRISMA Extension for Scoping Reviews (PRISMA-ScR) [45].

### Article eligibility criteria

#### Types of participants

Eligible studies will investigate adults (≥ 18 years) with CKD (defined as an estimated GFR < 60 mL/min/1.73 m^2^ for > 3 months [46]), including adults with ESKD treated with dialysis (all modalities). Studies will be included irrespective of the participants’ baseline cognitive status. Studies focusing on children or people living with kidney transplants will be excluded, as their needs for cognitive interventions might differ substantially from patients with CKD or ESKD on dialysis.

#### Concept

Cognition is defined by the American Psychological Association as “all forms of knowing and awareness, such as perceiving, conceiving, remembering, reasoning, judging, imagining, and problem solving [47].” A cognitive intervention will be defined as any intervention (pharmacological and/or non-pharmacological) that targets cognition, or ≥ 1 of the following specific cognitive domains included in the “mental function” component of the World Health Organization International Classification of Functioning [48]: consciousness, orientation, intellectual function, attention, memory, perception, thought functions, higher-level cognition (e.g., executive functions and problem solving), mental functions of language, calculation, and/or experience of self and time. Studies will also be included if they are investigating the impact of an intervention on cognition or ≥ 1 of the aforementioned cognitive domains, irrespective of the nature of the intervention. Studies will be excluded if they report approaches not specific to cognition, namely, cognitive behavioral interventions designed to only target mood or psychosocial well-being, but not cognition, and self-management interventions that are not aimed at specifically addressing cognitive impairment.

#### Context

Studies from all years, countries, and practice settings will be considered. Studies that are not available in English will be excluded.

### Types of sources

Information sources will include full-text, primary research articles and reports. Primary studies using qualitative, quantitative, or mixed methods designs (including randomized controlled trials, quasi-experimental studies, pre-post studies, observational studies, pilot studies, single-case experiments, and qualitative studies) will be included, with no limits placed on publication date. We will exclude case series, case studies, clinical practice guidelines, theoretical papers, theses, and opinion-driven reports (editorials, non-systematic or literature/narrative reviews). Scoping and systematic review articles will be examined during the initial search to identify additional eligible full-text articles from reference lists, but will otherwise be excluded.

### Search strategy and article selection

**Fig. 2:**
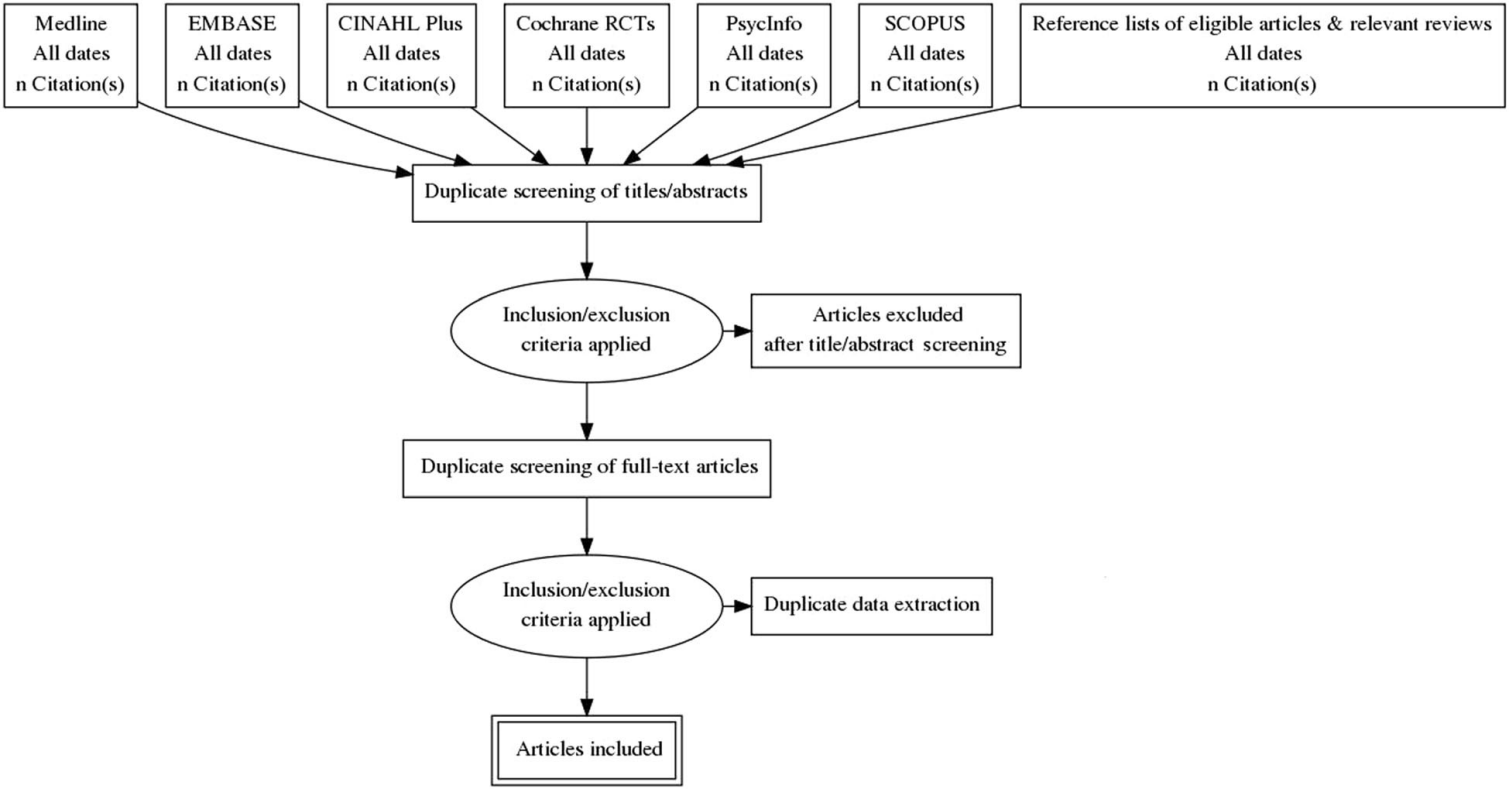
PRISMA flow diagram of study selection process

The screening process is outlined in the PRISMA diagram in Fig. 2. We worked with an information specialist to select search terms that represent the target population (CKD and concept (cognition)), as well as intervention-related terms (Table 1). Our selection of search terms was also informed by published reviews on cognitive interventions [36, 37]. We will search five electronic databases using the search terms, to identify relevant literature. These include MEDLINE (OVID), EMBASE, PsycINFO, Cochrane Central Register of Controlled Trials, and CINAHL Plus. We will also search Scopus to identify meeting abstracts and summaries. Finally, we will perform backward citation searching, which involves examining reference lists of included studies and relevant systematic/scoping reviews to identify missed literature. A search of grey literature will also be conducted using the Canadian Agency for Drugs and Technologies in Health (CADTH) guidelines. Specifically, we will use the CADTH recommendations to search online search engines, including Google Canada/US/UK, relevant health technology assessment agencies, and clinical trials databases, to identify additional research reports and sources relevant to the review.

**Table 1:** Review search terms

Population terms	Condition terms	Intervention terms
Chronic kidney disease Chronic kidney failure Chronic renal disease Chronic renal failure End-stage renal disease End-stage renal failure End-stage kidney disease End-stage kidney failure Dialysis H(a)emodialysis	Cognition Dementia Alzheimer Orientation Metacognition Consciousness Unconsciousness Attention Perception Memory Executive functioning Recall Problem solving Mental processing Thinking Comprehension Reasoning Judgment Reaction time Neuropsychological Neurocognitive Neuroprotective	Intervention Program Treatment Therapy Medication Drug Pill Rehabilitation Remediation

Following the initial search, results will be imported into Rayyan [49], an online tool designed to assist with and organize article screening. Two reviewers will screen all titles and abstracts independently in duplicate. In the case that total titles and abstracts are > 10,000, we will have three reviewers perform triplicate screening independently for 10% of titles and abstracts. The reviewers will convene after triplicate screening to discuss disagreements and clarify criteria. Inter-rater reliability will be calculated using the weighted Kappa statistic to ensure adequate agreement (defined as Kappa > 80). When Kappa > .80 is reached, the reviewers will commence screening independently. After title/abstract screening is complete, full-text screening will be conducted for all articles independently in duplicate. Any disagreements about eligibility will be resolved by discussion with a third reviewer, to obtain consensus.

### Charting, collating and summarizing data

A data extraction sheet has been developed a priori for the review, using reporting guidelines such as the Template for Intervention Description and Replication (TIDIER) [50] to ensure comprehensive data extraction. Two reviewers will perform independent data extraction for each included study, and disagreements about data extracted will be resolved by discussion with a third reviewer. Data to be extracted will include article characteristics (e.g., authors, date, journal, and study design), population characteristics (e.g., age, sex, CKD stage and definition, and screening criteria), cognitive intervention characteristics (e.g., aims, theoretical background, design, dose/duration, materials, location, provider(s)), and study outcomes (e.g., outcome measures used; results reported) (Table 2). The primary and secondary outcomes of each study will be categorized into one of eight health outcome types described previously [51], which include cognitions, behaviors, physiological measures, symptoms, health status, and healthcare. The reported findings of each outcome for each included study will then be described as positive, unchanged, or negative. Counts and percentages will be used to summarize patterns in the literature. A narrative summary of the main characteristics of the literature will then be conducted.

**Table 2:** Data extraction variables

Article	Title Year Authors Journal Country
Population	CKD definition/stage and treatment modality (if applicable) Mean/median age Sex Secondary diagnoses/health conditions Income (if given) Marital status Education Sampling method Eligibility (inclusion/exclusion) criteria Baseline cognitive status
Intervention	Aims Theoretical rationale Stakeholder involvement Materials Provider(s) Mode of delivery Procedures Setting Dose/duration Tailoring Treatment fidelity Withdrawal rate
Study and Results	Study design Control condition used Assessment time points Outcome measures used How outcomes examined (i.e., statistical approaches) Results (positive, negative, unchanged)

## Discussion and implications

Cognitive impairment is already a well-established problem in CKD, affecting an estimated 20–50% of people with moderate CKD and as many as two thirds of people on chronic dialysis [1, 6]. The breadth of literature on cognitive interventions in populations outside of CKD suggests there may be ways to mitigate cognitive impairment and its negative effects in the CKD population. However, the state of research into cognitive interventions in people with CKD has never been comprehensively explored. The potential negative implications associated with unaddressed cognitive impairment include a loss of independence [16, 52], prolonged hospitalization [53], institutionalization [18, 19], and mortality [15]. The economic costs of cognitive impairments such as dementia are also rapidly increasing, with estimates suggesting a global dementia cost of 818 billion US dollars in 2015 and a projected cost of approximately two trillion dollars by 2030 [54]. In chronic conditions such as CKD, unaddressed cognitive impairment may also result in disease mismanagement, medication errors, or other safety concerns. It is therefore a timely and pertinent initiative to obtain a complete understanding of what is known about cognitive interventions for CKD, to inform a research agenda for advancing knowledge in this area.

This review will systematically and comprehensively describe current research on pharmacological and non-pharmacological cognitive interventions in CKD. It will clarify the breadth of interventions that have been investigated in people with CKD and what is known about them, which can be used to highlight emerging areas in this field that warrant further investigation. It will contribute a critical examination of approaches to research and intervention thus far, examining whether elements such as the theoretical rationale of the intervention, study screening procedures, use of cognitive and functional outcome measures, and implementation potential and stakeholder perspectives have been adequately addressed. It will shed light on the transferability of established cognitive interventions (e.g., pharmacological agents, cognitive rehabilitation, and exercise) to the CKD population, and draw attention to unexplored approaches from other populations that can be pursued in future CKD research. It will also draw attention to literature on interventions unique to CKD or ESKD and its treatment (e.g., dialysis techniques) that have been shown to influence cognitive outcomes, providing nephrology practitioners with a summary of information that is relevant to the care of people with CKD who have cognitive impairments. Collectively, the knowledge gained from this review can be used help to enhance the quality and impact of cognitive intervention research in CKD in the future, and can inform future intervention systematic reviews in this area.

This review has several methodological strengths. We are adhering to the gold standard Joanna Briggs Institute guidance [44] to guide the conduct of the scoping review, which will help to maximize its quality and thoroughness. Both the protocol and the final manuscript will also adhere to the PRISMA-SCr reporting guidelines [45], which provide additional guidance on key features to address in a scoping review report. Our review features a comprehensive and systematic literature search, using a list of search terms informed by previous cognitive reviews and refined by an information specialist, which will help to ensure that all relevant full-text articles on this topic are identified. We are also conducting duplicate full-text screening and data extraction of eligible articles to maximize the trustworthiness of our findings.

The limitations of this review include those inherent to scoping review methodology, such as a lack of quality assessment or critical appraisal of included intervention-based articles, which will limit our ability to identify evidence gaps related to scientific limitations of studies. However, a scoping review is intended to uncover the breadth and extent of research that exists in field and can help to determine whether a systematic review that includes a quality assessment would be of value. Further, due to the large number of initial search results we expect to find, and resource limitations among our team, it may be that we are also unable to perform full duplication of title and abstract screening which could introduce a risk of inconsistent screening practices among screeners. However, we are addressing this limitation by conducting inter-rater screening validation for a subset of articles. We are excluding non-English studies from the review due to a lack of capacity to screen articles not available in English, which may limit the generalizability of our findings to non-English populations. Last, there is also the chance that we will miss some relevant cognitive-related data that is embedded only within the full-text of articles and not in the titles, abstracts, or keywords where databases search. It may be that some modalities or treatment approaches may secondarily influence cognitive outcomes, but these outcomes are not as easily identified by the structure of the published articles.

## Conclusion

This scoping review will provide a comprehensive description of research activity to date in the area of cognitive interventions for people with chronic kidney disease. Results will inform recommendations for future research that can advance the field in accordance with best practices from the broader cognitive intervention literature.

## Data Availability

The datasets used and/or analyzed during the current study are available from the corresponding author on reasonable request.

## Abbreviations

CKD: Chronic kidney disease
ESKD: End-stage kidney disease
JBI: Joanna Briggs Institute
PRISMA-ScR: Preferred Reporting Items for Systematic Reviews and Meta-Analyses–Scoping Reviews
